# Lifestyle and Health among Spanish University Students: Differences by Gender and Academic Discipline

**DOI:** 10.3390/ijerph9082728

**Published:** 2012-08-02

**Authors:** Verónica Varela-Mato, José M. Cancela, Carlos Ayan, Vicente Martín, Antonio Molina

**Affiliations:** 1 Faculty of Education and Sports Science, University of Vigo, Xunqueira Campus, Pontevedra 36005, Spain; Email: chemacc@uvigo.es (J.M.C.); cayan@uvigo.es (C.A.); 2 IBIOMED, University of Leon, Vegazana Campus, León 24400, Spain; Email: vmartins@telefonica.net (V.M.); ajmolina@unileon.es (A.M.)

**Keywords:** academic discipline, alcohol, eating disorders, health, gender, physical activity, Spain, tobacco, university

## Abstract

Today the need to analyze health behaviour from a gender perspective is as imminent as ever, particularly at university, where the number of women who register is on the rise and has exceeded the number of male students worldwide. We carried out a prevalence study aimed at analyzing Spanish university students’ lifestyles and identify differences according to gender and academic discipline. Of 3,646 eligible subjects doing university courses related to health (Group A), education (Group B) and other professions (Group C), 985 (27.0%) participated in the study. Information was elicited about their physical activity level, disturbed eating attitudes, consumption of alcohol, tobacco and illegal substances. Prevalence and Odds Ratios (OR) were calculated according to sex and kind of academic discipline. The obtained data confirmed that only 27.4% of the students were considered as sufficiently active, while 14.9% of them suffered from disturbed eating attitudes (DEA). Women were particularly less active (OR 0.46 (0.32–0.66); *p* < 0.0001), and more sedentary than men (OR 1.40 (1.00–1.97); *p* = 0.03). Binge drinking was more frequent in female than in male students (OR 1.79 (1.29–2.47); *p* = 0.0004). A third of the analyzed sample admitted that they had used illegal substances, while a lower consumption prevalence was found in women (OR 0.53 (0.40–0.71); *p* < 0.0001). The studied population was not very active (27.4%), especially women (OR = 0.45). Therefore, it seems that Spanish university students lead an unhealthy lifestyle, a situation which seems more conspicuous amongst females.

## 1. Introduction

Drug consumption, especially tobacco and alcohol, unsuitable diet and insufficient physical activity (PA) determine an important part of the deaths and illnesses that occur in the European region of the World Health Organization. These lifestyles are largely modifiable through political action, and it is interesting for public health to know their evolution and trends in different communities and population groups [1].

Among these groups, the health habits of university students are a special concern, since they represent a major segment of the young population and they are at a stage of their lives during which important lifestyle modifications take place [2]. If these changes become fixed routines, they are likely to determine the person’s future health [3,4]. This kind of research becomes especially necessary at university, since students make up a homogenous and accessible population, who also can generally be considered to be in relatively good health. The need to analyze health behaviour from a gender perspective has been pointed out by some authors [4,5]. The gender differences among university students in health are a consequence of different structural contexts for genders (age, social support, and family arrangement), lifestyle (smoking, drinking, exercise, diet) and psychosocial factors (critical life events, stress, and psychological resources) [6]. It has been noted by some authors that women’s health is more influenced by structural and psychosocial factors such as stress and lower levels of self-esteem and sense of coherence, while men’s health was more affected by health behaviours such as smoking, drinking and physical activity [6]. It has also been pointed out that it would be most convenient for the university population, especially for future health and education professionals, to develop and lead a healthy lifestyle, since they will be responsible for the encouragement of healthy habits among future generations [3]. This fact has fostered research on university students’ lifestyle, especially in those students who are doing a health-related university degree [7,8].

However, to the author’s knowledge, it seems that scientific research aimed to compare and identify healthy habits among university students, taking into account their gender and chosen academic discipline, are less frequent [9,10], especially in Spain [11]. Thus, the aim of the present study is to assess the lifestyle of Spanish university students in order to: (a) ascertain gender differences in healthy habits if they exist and (b) determine whether the academic discipline that the selected students take part in are related to those behaviours.

## 2. Experimental Section

### 2.1. Study Design and Sample

A transversal descriptive study was carried out including all students registered during the 2009/2010 academic year in the Pontevedra Campus of the University of Vigo (Spain). The 2009/2010 academic year was chosen, as it was the last period before the academic system went through adjustment due to the launch of the ‘European Higher Education Area’. The sample size was calculated with a 90% (1 – α) confidence level, of which the accuracy (d) will be 1%, the expected proportion will be 50% and the expected missing data will be 4%. The sample was divided into three groups according to the kind of university degree that the participants were studying (Table 1). Group A was composed of students who were training to become health professionals; Group B included students doing an education-related degree and Group C included those students registered in university degrees related to professional fields which differed from the aforementioned groups. Data collection took place during a regular class of the subject with the highest number of matriculated students for the course and the chosen university degree selected, to minimize losses. An “*ad hoc*” questionnaire was voluntarily and anonymously self-answered by the students. All participants gave their informed and written consent and received a small gift in appreciation for their time. The study obtained the approval of the university’s Students Vice-Chancellery and the government bodies of the involved academic centres. It was also assessed and approved by the Ethics Committee of the University of Vigo.

**Table 1:** Sample participation according to gender and academic discipline.

	Total			Women			Men		
	Matriculated	Participants		Matriculated	Participants		Matriculated	Participants	
	N	n	%	N	n	%	N	n	%
**Group A**	**821**	**288**	**35.10%**	**391**	**158**	**40.40%**	**430**	**130**	**30.20%**
Higher degree in physical education and sport	468	134	28.60%	124	39	31.50%	344	95	27.60%
Nursing	171	80	46.80%	147	65	44.20%	24	15	62.50%
Physiotherapy	182	74	40.70%	120	54	45.00%	62	20	32.30%
**Group B**	**1,233**	**318**	**25.80%**	**868**	**259**	**29.80%**	**365**	**59**	**16.20%**
Childhood Education	496	148	29.80%	418	145	34.70%	78	3	3.80%
Primary Education	341	87	25.50%	266	73	27.40%	75	14	18.70%
Physical Education	201	45	22.40%	67	16	23.90%	134	29	21.60%
Musical Education	195	38	19.50%	117	25	21.40%	78	13	16.70%
**Group C**	**1,592**	**379**	**23.80%**	**1,059**	**247**	**23.30%**	**533**	**132**	**24.80%**
Advertising and Public Relations	464	144	31.00%	355	109	30.70%	109	35	32.10%
Fine Arts	591	67	11.30%	414	42	10.10%	177	25	14.10%
Management and Public Administration	160	62	38.80%	88	44	50.00%	72	18	25.00%
Audio-visual Communication	170	59	34.70%	121	39	32.20%	49	20	40.80%
Forest Engineering	207	47	22.70%	81	13	16.00%	126	34	27.00%
**Total**	**3,646**	**985**	**27.00%**	**2,318**	**664**	**28.60%**	**1,328**	**321**	**24.20%**

### 2.2. Measurements

Physical activity level was assessed by means of the Spanish short version of the International Physical Activity Questionnaire (IPAQ) which has shown good reliability (r = 0.88) and moderate validity (ρ = 0.26) coefficients [12]. Considering the criteria of frequency, intensity and duration, the identified PA levels were classified in: Low (L), Moderate (M) and High (H). Physical activity was defined as “high”, according to current public guidelines recommended for healthy PA [13] and in line with other authors’ findings [14]. Partial and total scores are shown in METS-minutes/week (L < 600 METS-min/week; M < 1,500 METS-min/week and H ≥ 1,500 METS-min/week). Therefore, those students classified as “Low” undertook a sedentary lifestyle. However, those classified as “High” were considered as sufficiently active. 

The Spanish version of the Eating Attitudes Test (EAT-40) [15] was used to detect disturbances in eating behaviour and attitudes towards nutrition. A cut-off score of 21 was chosen given that it has been observed that the best diagnostic prediction was obtained around this value when screening for eating disorders in university students in a non-clinical setting [16]. The EAT-40 validity and reliability was conducted in Spain by Castro *et al*. [15] which obtained values of 0.68 and 0.91, respectively.

Self-reported tobacco smoking behaviours, drinking habits and consumption of toxic substances were assessed by means of a questionnaire specifically designed for this purpose in young Spanish populations [17]. Respondents were asked whether they consumed any amount of alcohol on a weekly basis (alcohol consumption). If so, data was elicited about their preferred alcoholic drink and the quantity of alcohol usually consumed. This was measured using the standard drink unit “SDU” [18]. According to standard health recommendations, binge drinking was defined as an intake of 8 or more SDU in men and 6 or more in women during any one sitting (one evening/night) during the preceding 30 days [19]. Tobacco consumption was assessed using the following question: Do you smoke? Participants could respond with either “I do not smoke”, “Sometimes, I smoke”, “I only smoke during the weekend” or “I smoke everyday”. Respondents who claimed to smoke were also required to state the average number of cigarettes that they smoked per day. In this regard, those who smoked at least one cigarette weekly were considered “usual smokers”. Finally, students were asked if they had ever used illicit drugs (illegal substances consumption). If so, they were to report the most commonly consumed drug.

### 2.3. Statistical Analysis

The sample distribution was carried out taking into account both gender and academic discipline. The obtained sample was compared with the original population with the aim to identify any possible bias in the data collection. The descriptive analysis was carried out based on relative frequencies and percentages. Prevalence was calculated with confidence intervals of 95% for each of the categories of the studied variables. In order to assess the association among the studied variables and the different categories, an Odds Ratio calculation (OR) was carried out with confidence interval of 95% as well as the Chi-Square Test. A stratified analysis according to gender and academic discipline was also carried out and the corrected OR’s for the studied prevalence were calculated by the Mantel-Haenszel test. The statistical computer software SPSS-16 was used to store, recode and proceed to the descriptive treatment of the data. For the epidemiological analysis, association measurements and stratified analysis the program EPINFO for Windows 3.5.1 was used.

## 3. Results and Discussion

### 3.1. Participation Analysis

Out of the 3,646 students (63.6% women, 36.4% men), based on the campus where the research was conducted, 1,055 agreed to participate in the survey, giving a total of 985 (67.4% women, 32.6% men), fully completed questionnaires. This assumed a participation rate of 93.4%, also fulfilling the statistical power criteria. The participation of female students was significantly higher than that of male students (28.6% *vs*. 24.2%; *p* = 0.03). Whilst analysing the response rate in function with the chosen academic discipline, some significant differences were also identified (*p* < 0.001). According to the stratified analysis, males and Group C students showed a significantly lower participation rate (*p* < 0.004; *p* < 0.001). The degree of participation according to gender academic discipline is shown on Table 1.

Among the participants, the proportion of obtained answers was higher than 90% for tobacco, alcohol and drugs based questions. However, the response rate was a lot lower for the IPAQ (67.4%) and EAT-40 (75.1%). Differences according to gender were ascertained since the rate of non-answered questions was significantly higher in women in the IPAQ (41.7% *vs*. 30.5%; *p* < 0.001), and lower in the EAT-40 (9.2% *vs*. 54.2%; *p* < 0.001). Regarding the chosen academic discipline, a higher rate of non-answered questions was observed in the EAT-40 in groups A and C compared to group B (26.7 and 27.4% *vs*. 17.3%; *p* = 0.002). However, in the stratified analysis significant differences were only identified in the percentage of non-answered questions according to gender. 

### 3.2. Main Results

The prevalence of sedentary lifestyle, intense PA, eating disorders and different toxic habits according to gender and student’s degree, can be observed in Table 2. The crude OR and adjusted OR according to the student’s degree are shown.

**Table 2:** Descriptive statistics of health-related behaviours analyzed by gender and crude OR and adjusted for student’s degree.

Variables	N	N	%	OR	Adjusted OR for Student’s Degree	*P*
**Sufficient physical activity (IPAQ > 1,500 METS-min/week)**						
Total	610	167	27.4			
Men	223	86	38.6	1	1	<0.0001
Women	387	81	20.9	0.42 (0.29–0.61)	0.46 (0.32–0.66)	
**Sedentary lifestyle (IPAQ < 600 METS-min/week)**						
Total	610	291	47.7			
Men	223	93	41.7	1	1	0.03
Women	387	198	51.2	1.46 (1.05–2.04)	1.40 (1.00–1.97)	
**Disturbed eatings attitudes (EAT > 20)**						
Total	739	110	14.9			
Men	147	12	8.2	1		0.006
Women	592	98	16.6	2.23 (1.19–4.19)	1.84 (0.97–3.51)	
**Alcohol consumption**						
Total	982	756	77.0			
Men	319	267	83.7	1	1	0.0007
Women	663	489	73.8	0.55 (0.39–0.77)	0.58 (0.41–0.83)	
**Binge drinking**						
Total	686	349	50.9			
Men ≥ 8 SDU	240	99	41.3	1	1	0.0004
Women ≥ 6 SDU	446	250	56.1	1.82 (1.32–2.50)	1.79 (1.29–2.47)	
**Tobacco consumption**						
Total	973	328	33.7			
Men	316	100	31.6	1	1	0.38
Women	657	228	34.7	1.15 (0.86–1.53)	1.06 (0.78–1.43)	
**Illegal substances consumption**						
Total	972	344	35.4			
Men	314	141	44.9	1	1	<0.0001
Women	658	203	30.9	0.55 (0.42–0.72)	0.53 (0.40–0.71)	

The obtained data confirmed that only 27.4% of the students could be considered as sufficiently active, whilst 14.9% of them suffered from disturbed eating attitudes (DEA). Women were especially less active (OR 0.46 (0.32–0.66); *p* < 0.0001), and more sedentary than men (OR 1.40 (1.00–1.97); *p* = 0.03). Besides, they showed a higher prevalence of DEA (OR 1.84 (0.97–3.51); *p* = 0.006).

Daily alcohol consumption was found to be low (1.5%), but the percentage of habitual consumers was high (77%), and significantly lower in women (OR 0.58 (0.41–0.83); *p* = 0.0007). However, binge drinking was more frequent in female than in male students (OR 1.79 (1.29–2.47); *p* = 0.0004). 

Regarding tobacco consumption, it seems that around a third of the students from both genders claimed to be usual smokers, without statistically significant differences between men and women (OR 1.06 (0.78–1.43); *p* = 0.38). In a similar pattern, approximately a third of the analyzed sample admitted that they had used illegal substances, while a lower consumption prevalence in women was found (OR 0.53 (0.40–0.71); *p* < 0.0001). No differences were observed regarding daily consumption (OR 0.56 (0.25–1.23); *p* = 0.22). The prevalence of healthy habits and their associations with gender and the course studied can be seen in Table 3. 

The crude OR and adjusted OR according to gender are shown. A significantly higher prevalence of intense PA was found on Group A when compared with Groups B and C (35.6% *vs*. 23.4% and 23.7%; *p* = 0.082), but no significant differences were observed regarding the prevalence of sedentary students (42.3% *vs*. 50.0 and 50.4; *p* = 0.185). As far as DEA are concerned, Group B showed a significantly higher prevalence (19.4%) than Group A (11.1%; *p* = 0.049) but no more than Group C (13.4%; *p* = 0.09), mainly due to the high number of men who claimed to suffer from these disorders (19.2%). No significant differences were found in the assessment of the association of academic discipline on alcohol consumption, but this was not the case regarding smoking, since Groups B and C showed higher prevalence than Group A (34.9% and 41.7% *vs*. 22.0%; *p* = 0.002 and *p* < 0.001, respectively). Regarding the consumption of illegal substances, significant differences were observed among the university degrees included in Group C, which showed higher prevalence than Groups A and B (44.1% *vs*. 29.4%; *p* < 0.001 and 30.6%; *p* = 0.005).

**Table 3:** Descriptive statistics of health-related behaviours analyzed by student’s degree and OR crude and adjusted for gender.

Variables		N	N	%		OR		OR Groups	Corrected OR
**Sufficient physical activity (IPAQ > 1,500 METS-min/week)**									
Group A		194	69	35.6				1	1
	Men	95	46	48.4		1			
	Women	99	23	23.2		0.32 (0.17–0.60)			
Group B		188	44	23.4				0.55 (0.35–087)	0.74 (0.46–1.18)
	Men	47	21	44.7		1			
	Women	141	23	16.3		0.24 (0.11–0.50)			
Group C		228	54	23.7				0.56 (0.36–0.86)	0.60 (0.39–0.93)
	Men	81	19	23.5		1			
	Women	147	35	23.8		1.02 (0.54–1.93)			
**Sedentary lifestyle (IPAQ < 600 METS-min/week)**									
Group A		194	82	42.3				1	1
	Men	95	31	32.6					
	Women	99	51	51.5		2.19 (1.22–3.93)			
Group B		188	94	50.0				1.37 (0.91–2.04)	1.17 (0.77–1.78)
	Men	47	19	40.4					
	Women	141	75	53.2		1.67 (0.86–3.27)			
Group C		228	115	50.4				1.39 (0.95–2.04)	1.33 (0.91–1.96)
	Men	81	43	53.1					
	Women	147	72	49.0		0.85 (0.49–1.46)			
**Disturbed eating attitudes (EAT > 20)**									
Group A		208	23	11.1				1	1
	Men	64	4	6.3		1			
	Women	144	19	13.2		2.28 (0.74–7.00)			
Group B		263	51	19.4				1.75 (1.11–2.77)	1.61 (1.02–2.54)
	Men	26	5	19.2		1			
	Women	237	46	19.4		1.01 (0.36–2.82)			
Group C		268	36	13.4				1.21 (0.74–1.98)	1.14 (0.69–1.86)
	Men	57	3	5.3		1			
	Women	211	33	15.6		3.34 (0.98–11.31)			
**Alcohol consumption**									
Group A		287	222	77.4				1	1
	Men	129	106	82.2		1			
	Women	158	116	73.4		0.60 (0.34–1.06)			
Group B		318	227	71.4				0.92 (0.84–1.01)	0.96 (0.88–1.06)
	Men	59	50	84.7		1			
	Women	259	177	68.3		0.39 (0.18–0.83)			
Group C		377	307	81.4				1.05 (0.97–1.14)	1.06 (0.98–1.15)
	Men	131	111	84.7		1			
	Women	246	196	79.7		0.71 (0.40–1.25)			
**Heavy episodic drinkers**									
Group A		206	96		46.6			1	1
	Men	97	39		40.2		1		
	Women	109	57		52.3		1.63 (0.94–2.83)		
Group B		209	110		52.6			1.27 (0.87–1.87)	1.11 (0.74–1.65)
	Men	48	21		43.8		1		
	Women	161	89		55.3		1.59 (0.83-3.04)		
Group C		271	143		52.8			1.27 (0.88–1.83)	1.19 (0.82–1.72)
	Men	95	39		41.1		1		
	Women	176	104		59.1		2.07 (1.25–3.44)		
**Tobacco consumption**									
Group A		286	63		22.0			1	1
	Men	129	27		20.9		1		
	Women	157	36		22.9		1.12 (0.64–1.98)		
Group B		315	110		34.9			1.59 (1.22–2.07)	1.55 (1.18–2.04)
	Men	58	19		32.8		1		
	Women	257	91		35.4		1.13 (0.61–2.06)		
Group C		372	155		41.7			1.89 (1.47–2.43)	1.88 (1.47–2.42)
	Men	129	54		41.9		1		
	Women	243	101		41.6		0.99 (0.64–1.52)		
**Illegal substances consumption**									
Group A		286	84		29.4			1	1
	Men	129	46		35.7		1		
	Women	157	38		24.2		0.58 (0.34–0.96)		
Group B		314	96		30.6			1.04 (0.81–1.33)	1.17 (0.90–1.51)
	Men	57	24		42.1		1		
	Women	257	72		28.0		0.54 (0.30–0.97)		
Group C		372	164		44.1			1.50 (1.21–1.86)	1.56 (1.27–1.94)
	Men	128	71		55.5		1		
	Women	244	93		38.1		0,49 (0.32–0.76)		

### 3.3. Discussion

This study aimed to analyze healthy habits in a Spanish university population, in order to identify students’ behaviour within their gender and chosen academic discipline. As university students, they are considered prospective professionals with important roles in the future. For this reason, their attitudes in terms of health behaviours it is of a heightened importance. It has been found that most of the students did not reach those levels established by the recommendations guidelines for health-enhancing PA (value of 1,500 METS/week) [20]. Although this is in fact in accordance with the general situation observed in Spain and in other countries [21,22]. In some cases, weekly sport practicing among students doing Health Science related degrees barely reached 60% among men and 35% among women [23]. For instance, moderate PA values have been obtained at 65% among physiotherapy students [24], and around 43%–50% among Nursery students [25]. The inactive attitude is especially worrying amongst women, since only 21% of the studied female population, exercised with adequate regularity and intensity. This is half percentage reported by men. Besides, while men who were studying a health-related university degree tended to be more active, PA level among women was similar in all degrees. It seems that there is a positive effect derived from the academic discipline. However, it did not have the same impact amongst males than in females and it seems that the most influenced by this particular variable are males doing health related degrees. However, we cannot establish a direct relationship between health behaviours and academic discipline, since we do not have the specific variable to describe it neither the results are definitive. In this regard, it should be mentioned that this sample follows the same pattern as students from 13 other different European countries, where males had a higher prevalence of physical activity practise, especially students related to health degrees [3]. Nevertheless, comparisons with other investigations are difficult to resolve, due to the different populations chosen as objects of the study, and the various ways of establishing a standardized PA level.

Regarding the analysis of eating disorders, our values seemed to be high, 8% for males and 17% for females, a fact which could be accounted for by the use of a cut-off score of 21. However, this cut-off point has been proposed as suitable for the study of Spanish young population [16]. Indeed, national and international studies that had used this very same cut-off point, have found a prevalence of DEA around 20%, similar to our findings. Nevertheless, the values were a bit different for females, in between 7.3–18%, and for males, 0.9–3% [11]. Finally, it is worth mentioning that the males’ values obtained from this sample are above than the normal expected for this gender.

As far as gender differences are concerned, the higher prevalence of unhealthy eating behaviours and attitudes among women is a well-known fact [26,27,28]. Furthermore, no significant differences have been found amongst females according to the academic discipline. Regarding men, those who were studying education-related degrees showed higher prevalence of DEA than the rest of the sample, though this result may be a spurious finding due to the bias caused by the low rate of answers obtained from that group. Yet this difference could derive from the fact that males regard DEA as something typically feminine and alien to their health habits.

Given these findings, we can establish that there is a need of health-promoting interventions to reduce these problems in the university environment, but fundamentally oriented to women. Thus, new tools should be researched for the assessment of DEA in males in order to improve the rate of answered questions and obtain more reliable results.

The present study shows a general high percentage of students who consumed alcohol (77%), especially at weekends and in high doses. No significant differences were found among students from the three different degrees, which is consistent with other studies on adolescents and university students in Spain [29,30]. In terms of binge drinking, and taking into account women’s different physiological characteristics, the observed proportion of heavy episodic drinkers was significantly higher among females than in males, 56% *vs*. 41%. This value could have been influenced by the sample distribution and by the fact that female university students tend to mirror the behaviours of their male peers regarding alcohol consumption [5]. Recent studies have also found higher percentages up to 63% for men and 83% for women in terms of binge drinking among university students [27]. Nonetheless, the lack of consistent criteria to define episodic drinkers [31,32], and the high cut-off point established for Spanish populations, limits the comparison of these findings across studies [33]. The relation between the academic discipline and alcohol use is not clear among university students from the three groups, since no significant differences were observed. Furthermore, a similar level of alcohol consumption was reported in other studies, where the influence of chosen university course seemed to have little or non-influence on their attitude towards alcoholic beverages [9,23,34].

In terms of tobacco consumption, our findings showed a slightly higher prevalence of tobacco use amongst university students, in comparison with other university populations from Spain. The mean value is superior than the results from two studies carried out amongst Medicine students (26.1%) and Physiotherapy and Nursing students (27.3%), in 2009 and 2011, respectively [35,36]. However, worldwide studies have reported a prevalence of smoking amongst university students with a similar range of consumption as we present in this study. In this regard, for male students the rate ranged between 23% and 44% and for females a more varied 9% to 46% [3]. 

Our results indicate a significant difference amongst students from the three different academic trainings. Thus, the lowest rate of smokers was reported in Group A, which is consistent with a tendency towards quitting tobacco observed in Spanish students doing health-related university degrees [8,37]. However, non-significant differences of tobacco consumption were observed between both genders. This could be interpreted as an indicator of the stage III in the tobacco epidemic, [38] which refers to a break in the habit of men and a global maintenance of the habit among women. It seems to be necessary to intervene this situation through prevention and controlling mechanisms fostered by academic training.

On the other hand, a high prevalence of illegal drugs use has indeed been identified, which is a common habit among university students [39]. The established significant differences, where the highest prevalence of consumption was reported by males and particularly by males from group C, follow previous reports [35]. However, the percentage of women who consumed illegal drugs cannot be considered as low, since a third percent of them appeared to take a drug of some kind. We have to take into account that similar patterns of consumption have been described by other studies, where the most popular drug was cannabis [9,40]. Furthermore, the fact that the lowest percentage of illegal drug use was reported by students from Group A can, to some extent, account for the association between tobacco and cannabis consumption described by several authors [41,42]. Therefore, tobacco consumption could be considered as a gateway for illegal drugs use, especially cannabis. Subsequently, a positive linear regression could be identified between the two variables, but the influence of this correlation in this sample is not clear. Future investigations should continue exploring this correlation and targeting interventions to reduce drugs use in the university setting.

To sum up our findings, it seems that the survey confirms previous reports where the university population show high prevalence of unhealthy habits. In general our students showed a very low level of PA and a high percentage of them could be identified as heavy episodic drinkers. Besides, high levels of tobacco use and illicit drugs were reported. However, students of health-related undergraduate degrees appeared to be more active, smoke less and use illegal substances less frequently. Although different health patterns have been observed between students from the three different groups, a firm association between the academic discipline and the higher or lower prevalence of healthy habits cannot be made due to the cross-Emailal design of the study. Furthermore, it is not known if such differences are due to the selected academic discipline alone or due to additional external factors. Our obtained results do however allow us to establish that university education does not provide a sufficient stimulus to encourage a healthy lifestyle amongst its students. Moreover, clear differences have been found regarding the habits of males and females students, which could be accounted for/by gender differences in aspects like dealing with critical life events, stress, and psychological resources, to which females are more sensitive than males [6].

Given all of the above, implementing new actions to promote a healthy lifestyle are required to a significant extent. This can be achieved through programs oriented towards an increased knowledge of the risk and consequences of unhealthy behaviours, but especially by encouraging a change in attitudes towards lifestyles at this pivotal stage in young people's lives. According to our findings, to be able to achieve a successful reduction in the prevalence of unhealthy habits, these strategies must incorporate gender differences, and be campus-wide whilst strongly focusing on faculties and schools themselves.

### 3.4. Study Strengths and Limitations

To our knowledge, this is the first study of its kind which has set out to analyse the influence of academic training on university students’ lifestyle by means of comparing health, education and other university courses. Since it covers a wide variety of health habits and it is based on a considerable sample size, this research extracts some interesting information from university students’ lifestyle in Spain. Despite this strength, there are a number of limitations that must be considered when interpreting the results of this study. First, relying on cross-Emailal data constrains the study’s ability to make causal inferences about the relationship found in the research. Secondly, the study sample was not randomly selected, and it may have not been representative of all the university students of the region in which the research was carried out, since only those who attended class were who answered the questionnaires. Therefore, caution needs to be exercised when attempting to generalize to other contexts or populations. Third, all findings were derived from a self-reported survey which may has some sort of bias, since participants may not be reliable in the report of their behaviours. Finally the different response rate obtained across the different targeted courses raises concern about generalizing the results of this study. 

## 4. Conclusions

Physical inactivity, smoking and alcohol consumption are risk factors found to significant extent amongst Spanish university students. This situation seems to be more conspicuous in women. Although there have been clear differences established in terms of lifestyle across academia disciplines, it hasn’t been possible to show a firm association between the two variables. The findings of this study clearly state that there is a need to foster gender-sensitive strategies to promote a healthy lifestyle among university students. 
