# *Correction*: Varela-Mato, V.; Cancela, J.M.; Ayan, C.; Molina, A.; Martín, V. Lifestyle and Health among Spanish University Students: Differences by Gender and Academic Discipline. *Int. J. Environ. Res. Public Health* 2012, *9*, 2728–2741

**DOI:** 10.3390/ijerph10083590

**Published:** 2013-08-13

**Authors:** Verónica Varela-Mato, José M. Cancela, Carlos Ayan, Antonio Molina, Vicente Martín

**Affiliations:** 1 Faculty of Education and Sports Science, University of Vigo, Xunqueira Campus, Pontevedra 36005, Spain; E-Mails: chemacc@uvigo.es (J.M.C.); cayan@uvigo.es (C.A.); 2 IBIOMED, University of Leon, Vegazana Campus, León 24400, Spain; E-Mails: ajmolina@unileon.es (A.M.); vmartins@telefonica.net (V.M.)

The authors wish to make the following correction to this paper [1]. In the article it is mentioned that “the study obtained the approval of the university’s Students Vice-Chancellery and the government bodies of the involved academic centers and also by the Ethics Committee of the University of Vigo.” However, it should read: “The study obtained the approval of the university’s Students Vice-Chancellery and the government bodies of the involved academic centers” *only*.

The authors apologize for the inconvenience.

## References

[1] Varela-Mato V., Cancela J.M., Ayan C., Molina A., Martín V. (2012). Lifestyle and health among Spanish university students: Differences by gender and academic discipline. Int. J. Environ. Res. Public Health.

